# Electrochemical sensing of sildenafil in pharmaceutical formulations using an (S,N)-doped reduced graphene oxide-modified electrode

**DOI:** 10.3762/bjnano.17.74

**Published:** 2026-08-11

**Authors:** Nguyen Quang Man, Ngo Thi Thanh Xuan, Vo Thi Khanh Ly, Le Van Thanh Son, Le Duc Anh, Le Thi Hong Phong, Nguyen Hai Phong, Pham Khac Lieu, Le Lam Son, Dinh Quang Khieu

**Affiliations:** 1 University of Medicine and Pharmacy, Hue University, 530000, Vietnam; 2 University of Sciences, Hue University, 530000, Vietnam; 3 Drug, Cosmetic, and Food Quality Control Center of Hue city, 530000, Vietnam; 4 University of Education and Science, The University of Danang, 550000, Vietnam; 5 Viet Nam National Institute of Occupational Safety and Health, Hanoi, Vietnam; 6 Institute of Materials Sciences, Vietnam Academy of Science and Technology, 18 Hoang Quoc Viet, Cau Giay, Hanoi City, 100000, Vietnam

**Keywords:** differential pulse voltammetry, electrode modifier, rGO, (S,N)-rGO, sildenafil

## Abstract

In this work, (S,N)-co-doped reduced graphene oxide ((S,N)-rGO) was successfully synthesized via thermal treatment and thoroughly characterized by X-ray diffraction, high-resolution transmission electron microscopy, elemental mapping, photoluminescence, and Raman spectroscopy, confirming the successful incorporation of nitrogen and sulfur into the graphene framework. The obtained (S,N)-rGO was used to modify a glassy carbon electrode for the electrochemical determination of sildenafil (SDN). The modified electrode exhibited enhanced electrocatalytic activity toward SDN oxidation, providing a well-defined current response. Under optimized conditions, the sensor showed a good linear relationship between peak current and SDN concentration in the range of 0.25–4.98 µM, with a low limit of detection of 0.24 µM and a limit of quantification of 0.78 µM. The proposed sensor demonstrated satisfactory repeatability, reproducibility, and stability, as well as good selectivity in the presence of potential interfering substances. The applicability of the method was validated by the determination of SDN in pharmaceutical samples. The results were in good agreement with those from high-performance liquid chromatography, indicating that the proposed electrochemical method is reliable and suitable for practical analysis.

## Introduction

Sildenafil (SDN) is a widely prescribed phosphodiesterase type 5 (PDE-5) inhibitor used for the treatment of erectile dysfunction and pulmonary arterial hypertension. Its pharmacological activity is based on the selective inhibition of PDE-5, which increases the intracellular level of cyclic guanosine monophosphate (cGMP), resulting in smooth muscle relaxation and vasodilation. Phosphodiesterase-5 inhibitors, including SDN, are considered first-line therapeutic agents and have demonstrated significant efficacy in improving vascular function and clinical outcomes [1]. Furthermore, these agents enhance nitric oxide-mediated signaling pathways, thereby promoting increased blood flow in vascular tissues [2]. The widespread clinical use of SDN has led to a substantial increase in global consumption, which is accompanied by growing concerns regarding its misuse and illegal adulteration. Recent studies have reported that PDE-5 inhibitors are frequently detected as undeclared active ingredients in dietary supplements and counterfeit pharmaceutical products marketed as “natural” remedies [3]. This situation poses a serious public health risk as such products often evade regulatory control and may contain unpredictable or excessive dosages.

To date, various analytical techniques have been developed for the determination of SDN. Conventional methods such as gas chromatography/mass spectrometry (GC/MS) [4], liquid chromatography–tandem mass spectrometry (LC–MS/MS) [5], and high-performance liquid chromatography (HPLC) [6–7] are widely employed because of their high accuracy and sensitivity. In addition, advanced spectroscopic and hybrid approaches, including surface-enhanced Raman scattering (SERS) using Ag/UiO-66-decorated porous structures [8], thin-layer chromatography coupled with UV–SERS detection (TLC–UV–SERS) [9], dual-labeled probe time-resolved fluorescence immunochromatography assay [10], and fluorescence-based methods [11], have also been reported. Despite their excellent analytical performance, these techniques generally require sophisticated instrumentation, complex sample preparation, and relatively long analysis time.

In this context, electrochemical sensing has emerged as a promising alternative for SDN determination due to its inherent advantages, including operational simplicity, rapid response, low cost, and high sensitivity [12–13]. These features make electrochemical methods particularly suitable for on-site monitoring and routine analysis [14–15]. Importantly, the electrochemical oxidation of SDN can be effectively enhanced by tailoring the electrode surface with functional nanomaterials, thereby improving both sensitivity and selectivity.

Among various nanomaterials, graphene-based materials, especially reduced graphene oxide (rGO), have attracted considerable attention as electrode modifiers due to their excellent electrical conductivity, large specific surface area, and favorable electrocatalytic properties [16–17]. However, pristine rGO still suffers from a limited number of active sites and insufficient selectivity toward specific analytes. To overcome these limitations, heteroatom doping has been proposed as an effective strategy to modulate the electronic structure and enhance catalytic activity [18–19]. In particular, nitrogen and sulfur co-doping has been demonstrated to significantly improve electrochemical performance by introducing defects, redistributing charge density, and facilitating electron transfer processes [20–21].

In our previous study [22], nitrogen- and sulfur-doped reduced graphene oxide ((S,N)-rGO) was explored as a potential electrode modifier for the electrochemical detection of SDN, showing promising electrocatalytic behavior. However, that work was mainly limited to a proof-of-concept study and lacked a systematic investigation of the sensing mechanism, optimization of experimental conditions, and a comprehensive evaluation of analytical performance. Although various electrochemical sensors have been reported for SDN determination, many of them rely on multicomponent nanocomposites, noble-metal nanoparticles, or relatively complicated fabrication procedures. In contrast, the proposed (S,N)-rGO-modified electrode is prepared through a simple and facile synthesis strategy while providing enhanced electrocatalytic activity arising from the synergistic effects of nitrogen and sulfur co-doping. Together with its satisfactory analytical performance and successful application to pharmaceutical formulations, these features constitute the main novelty and practical advantage of the present work. To address these limitations, the present study builds on our previous findings and develops a more systematic electrochemical sensing strategy. In this work, (S,N)-co-doped reduced graphene oxide ((S,N)-rGO) was synthesized via a facile thermal treatment using ammonium persulfate as a dual heteroatom precursor. Incorporating nitrogen and sulfur heteroatoms is expected to generate abundant active sites, enhance electron transfer kinetics, and improve adsorption affinity for SDN molecules. The synthesized material was used to modify a glassy carbon electrode (GCE) for electrochemical detection. The fabricated (S,N)-rGO/GCE sensor exhibited significantly enhanced electrocatalytic activity toward the oxidation of SDN, resulting in improved sensitivity and selectivity compared with the bare electrode. The effects of key experimental parameters, including pH, scan rate, and accumulation conditions, were systematically investigated to elucidate the sensing mechanism. Furthermore, the analytical performance of the proposed sensor was thoroughly evaluated in terms of linear range, limit of detection, reproducibility, and stability. Finally, the practical applicability of the method was demonstrated by determining SDN in real pharmaceutical samples, with satisfactory accuracy and recovery.

## Experimental

### Chemicals

Graphite powder, phosphoric acid (H_3_PO_4_, 85%), sulfuric acid (H_2_SO_4_, 98%), hydrochloric acid (HCl, 37%), potassium permanganate (KMnO_4_, 99%), thiourea (CH_4_N_2_S, 99%), and hydrogen peroxide (H_2_O_2_, 30%) were obtained from Merck. Boric acid (H_3_BO_3_, 99.5%), phosphoric acid (H_3_PO_4_, 85%), acetic acid (CH_3_COOH, 99.8%), and sodium hydroxide (NaOH, 98%) were purchased from Sigma-Aldrich and used for the preparation of Britton–Robinson (BR) buffer solutions with various pH values for electrochemical measurements. SDN citrate standard (C_28_H_38_N_6_O_11_S, 99%) was supplied by the National Institute of Drug Quality Control, Vietnam. All chemicals and reagents used throughout the study were of analytical grade and utilized without any further purification.

### Apparatus

A conventional three-electrode system was used, consisting of a modified GCE as the working electrode, a platinum wire as the counter electrode, and an Ag/AgCl electrode as the reference electrode (CPA–HH5 Computerized Polarography Analyzer, Vietnam). Morphological and structural characterizations were performed using transmission electron microscopy (TEM) and energy-dispersive X-ray spectroscopy (EDX) mapping (JEM-2100, Jeol-Japan) at 200 kV, X-ray diffraction (XRD) (Bruker D8 Advance, Germany), Raman spectroscopy (XploRA™ PLUS, Horiba, Japan), FTIR (Shimadzu Prestige-21, Japan), and photoluminescence (PL) (Horiba Fluorolog 3 FL3-22 fluorometer).

### Synthesis of graphene oxide

Graphene oxide (GO) was synthesized following a modified procedure based on the method reported by Diego C. Marcano and coworkers [23]. Briefly, 3 g of graphite powder was gradually added to a mixed acid solution containing 360 mL of concentrated H_2_SO_4_ and 40 mL of concentrated H_3_PO_4_ under continuous stirring. Subsequently, 18 g of KMnO_4_ was slowly introduced into the mixture, resulting in a mildly exothermic reaction. The suspension was maintained under stirring for 72 h to allow for complete oxidation. Afterward, 17 mL of 30% H_2_O_2_ was added dropwise to terminate the reaction, and the mixture was allowed to cool to room temperature. The resulting product was separated by centrifugation at 5000 rpm for 15 min, and the supernatant was discarded to obtain a brownish-yellow solid. The solid was washed several times with 0.1 M HCl solution and deionized water to remove residual ions and impurities, followed by centrifugation. The purified material was then dried at 60 °C for 48 h to yield graphite oxide. This product was subsequently exfoliated by ultrasonic treatment in distilled water for 4 h. After centrifugation and drying at 60 °C for an additional 48 h, GO was obtained as a black solid.

### Synthesis of (S,N)-doped graphene oxide ((S,N)-GO)

The synthesis of nitrogen and sulfur co-doped graphene oxide ((S,N)-GO) was carried out using a procedure similar to that described above. The key difference lies in the oxidation stage, where 1.5 g of thiourea was introduced into the reaction mixture immediately after the addition of KMnO_4_, serving as a heteroatom dopant source.

### Synthesis of (S,N)-doped reduced graphene oxide

To obtain reduced (S,N)-doped graphene oxide ((S,N)-rGO), 0.3 g of (S,N)-GO was dispersed in a mixture of 50 mL distilled water and 30 mL ethanol. The suspension was ultrasonicated for 60 min to ensure uniform dispersion, then transferred into a Teflon-lined autoclave. The hydrothermal reduction was conducted at 180 °C for 12 h. After naturally cooling to room temperature, the resulting black solid was collected by centrifugation, washed thoroughly with distilled water, and dried at 80 °C for 24 h. The final product was ground into a fine powder to obtain (S,N)-rGO.

### Fabrication of the (S,N)-rGO-modified electrode

Prior to modification, the GCE surface was polished with an alumina slurry, thoroughly rinsed with deionized water, and sonicated sequentially in ethanol and water. A suspension of (S,N)-rGO (1 mg·mL^−1^) was prepared by dispersing the material in water with ultrasonication. A defined volume of the suspension (5 µL) was drop-cast onto the cleaned GCE surface and dried at room temperature (≈30 min) to obtain the (S,N)-rGO/GCE.

### Electrochemical measurements

The electrochemical behavior of SDN was investigated using cyclic voltammetry (CV) and differential pulse voltammetry (DPV) in a Britton–Robinson buffer solution at various pH values. Key experimental parameters, including accumulation potential, accumulation time, pulse amplitude, and scan rate, were optimized to achieve the best analytical performance. Calibration curves were constructed by measuring the peak current response at different concentrations of SDN. The limit of detection (LOD) and the limit of quantification (LOQ) were calculated based on the residual standard deviation of the calibration curve and its slope.

### Sample preparation and analysis

#### Sample preparation

Ten SDN tablets were accurately weighed and the average tablet mass was determined. The tablets were then finely ground to obtain a homogeneous powder. An accurately weighed portion of the powder, equivalent to one tablet, was transferred into a 100 mL volumetric flask. Approximately 50 mL of distilled water was added, and the mixture was sonicated for 30 min to ensure complete extraction of SDN. The solution was then diluted to the mark with distilled water, thoroughly mixed, and filtered to obtain the stock sample solution. The obtained solution was further diluted with distilled water to an appropriate concentration depending on the SDN content in the pharmaceutical formulation (solution 1). An aliquot of solution 1 was then diluted with distilled water and Britton–Robinson buffer (0.1 M, pH 4.0) in the electrochemical cell to obtain a final SDN concentration of approximately 4.2 µM. The SDN content was determined using DPV with the standard addition technique.

#### Recovery study

For recovery evaluation, the prepared sample solution was diluted under the same conditions as described above. Subsequently, 20 µL of a 0.001 M SDN standard solution was added into a 20 mL electrochemical cell. The total SDN concentration was determined by DPV using the standard addition method. The original SDN content in the sample was subtracted, and the recovery was calculated by comparing the measured increase with the known added amount. All measurements were performed in triplicate. The recovery (%) was calculated according to the following equation:




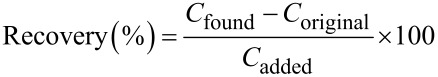




The relative standard deviation (RSD, %) was used to evaluate precision:




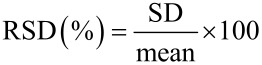




## Results and Discussion

### Characterization of materials

**Figure 1:**
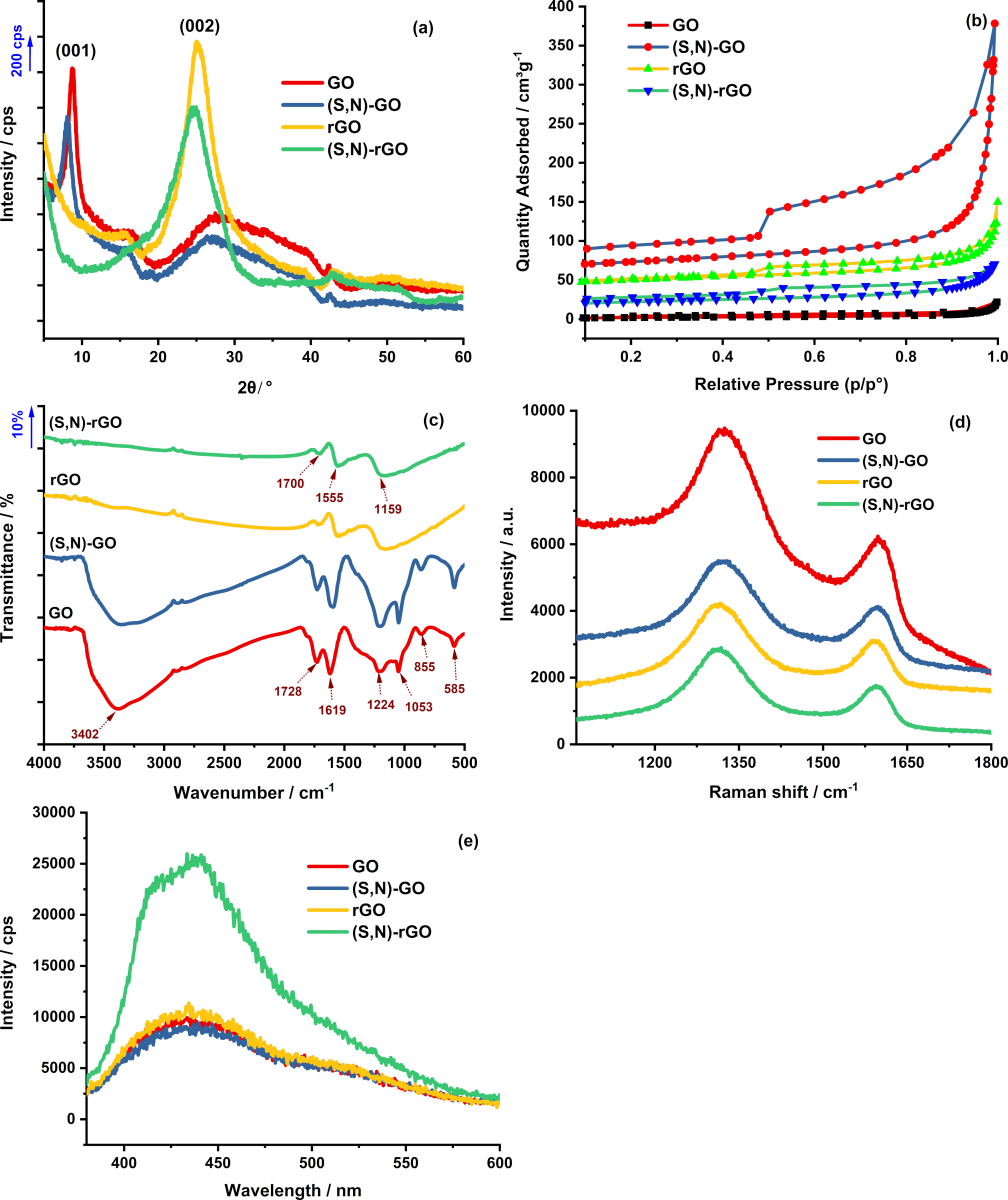
(a) XRD patterns, (b) nitrogen adsorption/desorption isotherms, (c) FTIR spectra, (d) Raman spectra, and (e) photoluminescence (PL) spectra of GO, (S,N)-GO, rGO and (S,N)-rGO.

Figure 1 presents the characterization results for the obtained materials. The GO sample exhibits a characteristic diffraction peak at around 2θ ≈ 8.7°, corresponding to the (001) plane, indicating the presence of oxygen-containing functional groups and enlarged interlayer spacing [24]. After (S,N)-doping, the peak becomes broader and slightly shifted, suggesting partial disruption of the layered structure. For (S,N)-rGO, the disappearance of the GO peak and the emergence of a broad peak at ≈24.7° (002 plane) confirm the successful reduction process and partial restoration of graphitic domains [24]. The textural parameters in Table 1 and Figure 1b reveal a clear evolution of pore structure after doping and reduction. Pristine GO exhibits a very low BET surface area (6.93 m^2^·g^−1^) with no detectable microporosity, indicating a highly stacked structure with limited accessible surface. Upon (S,N)-co-doping, (S,N)-GO shows a significant increase in surface area (63.81 m^2^·g^−1^), mainly contributed by mesopores, suggesting that the introduction of heteroatoms and gas evolution during oxidation promotes exfoliation and pore formation. After reduction, rGO presents a hierarchical pore structure with both micropores (16.5 m^2^·g^−1^) and mesopores (43.8 m^2^·g^−1^). The formation of micropores can be attributed to the removal of oxygen-containing functional groups, which generates vacancy defects and creates nanoscale pores within the graphene sheets. Meanwhile, mesopores originate from the partial restacking and wrinkling of graphene layers. In contrast, (S,N)-rGO shows reduced microporous (13.1 m^2^·g^−1^) and mesoporous (29.6 m^2^·g^−1^) contributions compared to rGO. This decrease may result from structural rearrangement or partial pore collapse during hydrothermal treatment, as well as the occupation of pore sites by doped nitrogen and sulfur species.

**Table 1:** Textural properties and Raman intensity ratios (*I*_D_/*I*_G_) of GO, (S,N)-GO, rGO, and (S,N)-rGO.

Sample	BET (m^2^·g^−1^)	Micropores (m^2^·g^−1^)	Mesopores (m^2^·g^−1^)	*I*_D_/*I*_G_

GO	6.93	–	6.9	0.82
(S,N)-GO	63.81	–	63.8	0.98
rGO	60.31	16.5	43.8	1.05
(S,N)-rGO	42.63	13.1	29.6	1.21

The FTIR spectra (Figure 1c) confirm the successful oxidation and subsequent modification of GO. The broad band at ≈3400 cm^−1^ corresponds to O–H stretching vibrations, while peaks at ≈1728 cm^−1^ (C=O), ≈1619 cm^−1^ (C=C), and 1224–1053 cm^−1^ (C–O) are characteristic of oxygen-containing functional groups in GO [25]. After doping and reduction, the intensity of oxygen-related peaks decreases, indicating partial removal of oxygen functionalities [24]. New features observed at ≈1555 cm^−1^ and ≈1159 cm^−1^ suggest the incorporation of nitrogen- and sulfur-containing groups, confirming successful heteroatom. The Raman spectra (Figure 1d) display the typical D and G bands of graphene-based materials [26]. The *I*_D_/*I*_G_ ratio increases from 0.82 (GO) to 1.21 ((S,N)-rGO), indicating an increase in structural defects and disorder after doping and reduction [27]. This increase is commonly attributed to the formation of smaller sp^2^ domains and the introduction of defects by N and S heteroatoms. Such defects are beneficial for electrochemical applications, as they enhance electron transfer and provide more active sites. The combined results confirm that (S,N)-co-doping and reduction effectively modify the structural, textural, and electronic properties of GO, leading to enhanced electrochemical activity. These structural and surface modifications are expected to significantly improve the electrochemical performance of the (S,N)-rGO-modified electrode.

The PL spectra of GO, (S,N)-GO, rGO, and (S,N)-rGO are shown in Figure 1e. All samples exhibit a broad emission band centered around 440–460 nm, which is commonly attributed to radiative recombination associated with oxygen-containing functional groups and defect states in graphene oxide-based materials. GO exhibits the highest PL intensity, indicating a high density of oxygenated defects that act as recombination centers for electron–hole pairs. After reduction and heteroatom doping, the PL intensity decreases significantly, especially for (S,N)-rGO. This quenching effect suggests the partial restoration of the conjugated sp^2^ carbon network and improved charge mobility after removal of oxygen-containing groups. The reduced PL intensity of (S,N)-rGO indicates a lower recombination rate of charge carriers and more efficient electron transport capability. In addition, the incorporation of nitrogen and sulfur atoms can modify the electronic structure of graphene and introduce new electronic states, thereby facilitating charge-transfer processes. The PL results therefore further confirm the successful reduction and heteroatom functionalization of GO, in agreement with the XRD, FTIR, BET, and Raman analyses.

**Figure 2:**
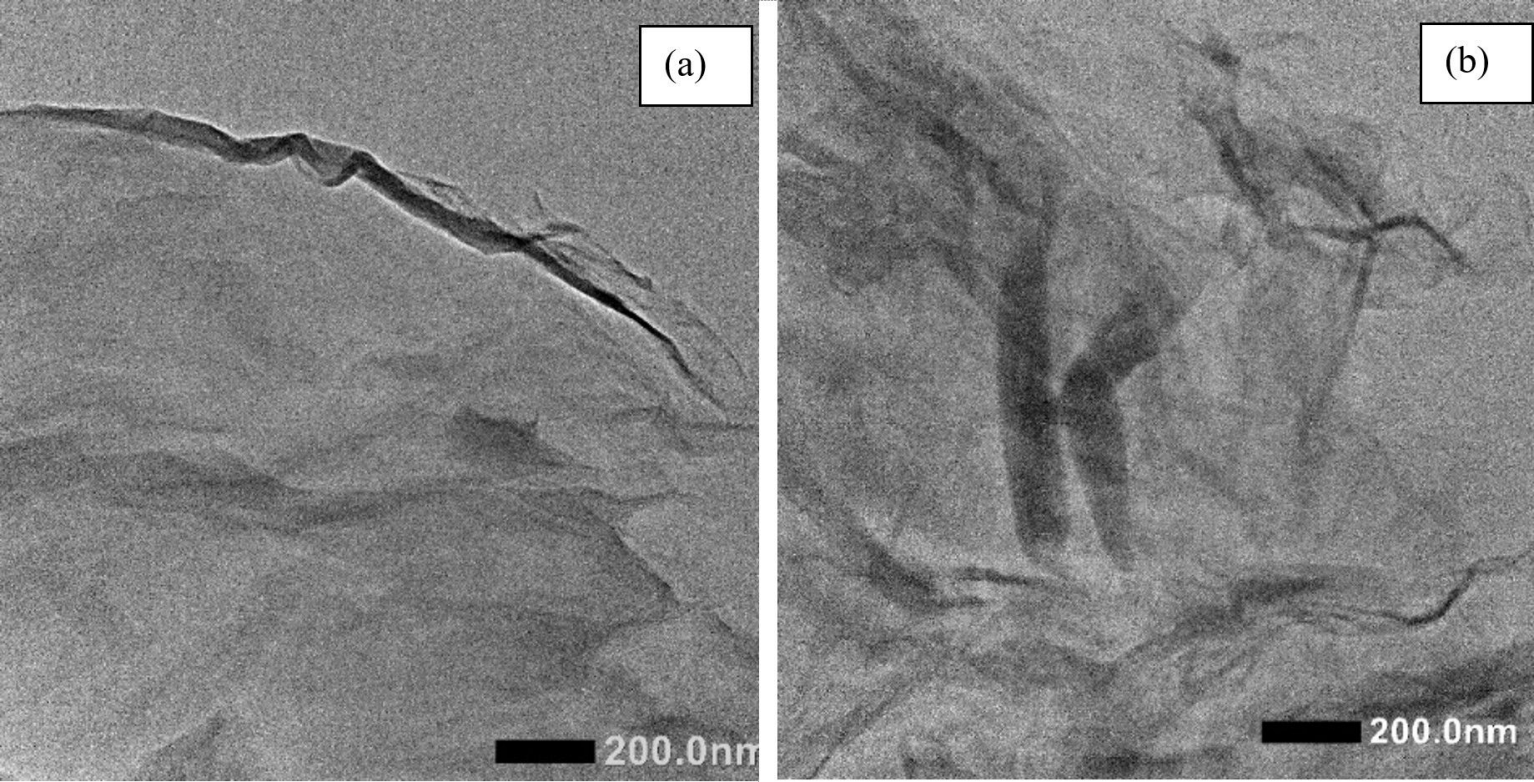
HRTEM observation of (S,N)-GO (a) and (S,N)-rGO (b).

High-resolution transmission electron microscopy (HRTEM) images of (S,N)-GO and (S,N)-rGO are presented in Figure 2a and Figure 2b, respectively. The (S,N)-GO sample exhibits thin, transparent, and relatively smooth layered nanosheets with a typical wrinkled morphology characteristic of graphene oxide-based materials. The translucent appearance indicates the formation of few-layer graphene sheets with high exfoliation degree. After the reduction process, the morphology of (S,N)-rGO becomes more crumpled and corrugated, accompanied by partial restacking of graphene layers. The increased wrinkling and folding observed in Figure 2b can be attributed to the removal of oxygen-containing functional groups during reduction, which induces structural distortion and decreases electrostatic repulsion between graphene sheets. Such morphological changes are commonly observed in reduced graphene materials and indicate the partial restoration of the conjugated carbon network. Moreover, the highly wrinkled and defective structure of (S,N)-rGO is advantageous for electrochemical applications because it can prevent severe agglomeration of graphene sheets, expose more electroactive surface area, and facilitate electrolyte diffusion. The formation of folded nanosheet structures also creates abundant edge-plane-like defects and active sites, which are beneficial for electron-transfer reactions and analyte adsorption during electrochemical sensing.

**Figure 3:**
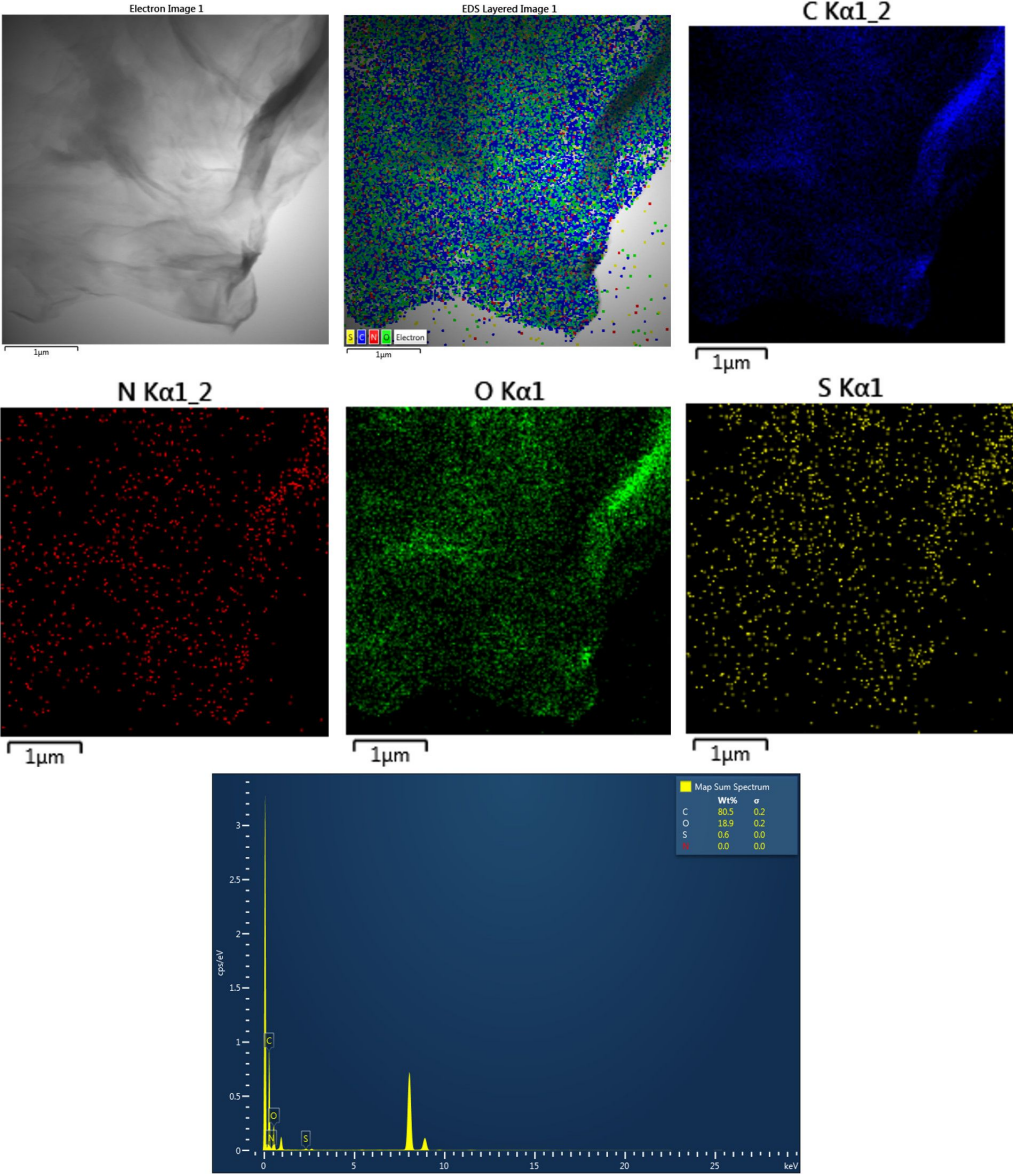
EDX mapping of (S,N)-rGO.

The elemental mapping images and EDX spectrum of (S,N)-rGO are presented in Figure 3. The mapping results reveal the distribution of C, O, N, and S throughout the graphene framework, confirming the successful heteroatom functionalization of the reduced graphene oxide sheets. The strong carbon signal originates from the graphene backbone, while the oxygen signal indicates that a small amount of residual oxygen-containing functional groups remains after the reduction process. Notably, although the quantitative EDX analysis shows an extremely low nitrogen content (close to 0 atom %), the elemental mapping image still exhibits detectable N-related signals distributed over the graphene surface. This discrepancy can be attributed to the inherent limitation of EDX spectroscopy in quantitatively detecting light elements at ultralow concentrations. Because nitrogen has a low atomic number and weak X-ray emission intensity, its signal can easily fall below the reliable quantitative detection threshold, particularly in carbon-rich materials such as graphene derivatives [28–29]. Nevertheless, the mapping image qualitatively suggests the presence of trace nitrogen species distributed over the graphene surface. However, owing to the limited sensitivity of EDX for the quantitative analysis of light elements, particularly nitrogen in carbon-rich materials, this observation should be regarded as qualitative evidence rather than definitive proof of nitrogen incorporation. Therefore, the presence of nitrogen-containing species is suggested but cannot be conclusively confirmed by EDX alone. In contrast, sulfur signals are more clearly observed and uniformly distributed across the graphene sheets, indicating more effective sulfur incorporation into the carbon framework.

### Determination of SDN using a (S,N)-rGO modified electrode

#### Electrochemical behavior of SDN on different electrode

**Figure 4:**
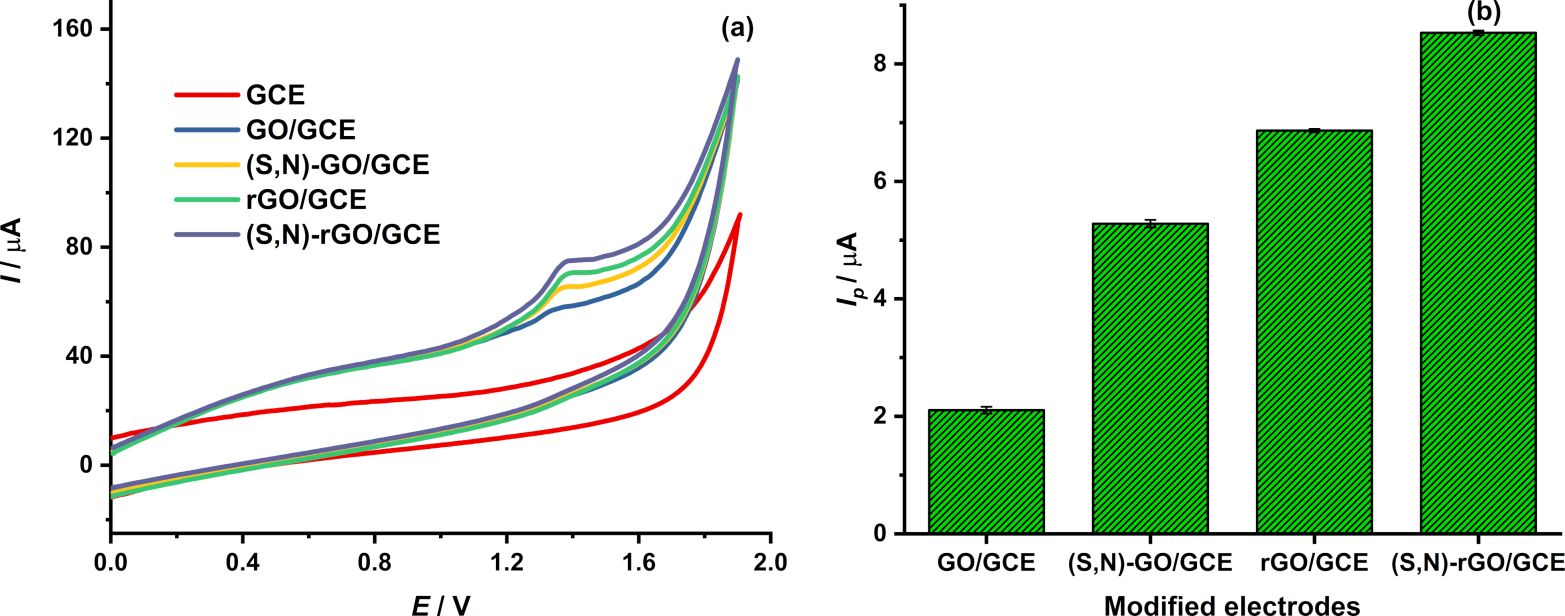
(a) Cyclic voltammograms (CVs) recorded at different electrodes (GCE, GO/GCE, (S,N)-GO/GCE, rGO/GCE and (S,N)-rGO/GCE) in 0.1 M BR buffer (pH 3.0) containing 20 µM SDN at a scan rate of 0.2 V·s^−1^; (b) corresponding peak currents obtained at different electrodes (*n* = 3).

The electrochemical responses of SDN at different electrodes are presented in Figure 4a. The bare GCE exhibits a relatively weak oxidation current, indicating limited electron transfer kinetics. Upon modification with GO, the peak current increases slightly due to the larger surface area and presence of oxygen-containing functional groups. A more pronounced enhancement is observed for the (S,N)-GO/GCE, suggesting that heteroatom doping improves the electrical conductivity and introduces additional active sites for electron transfer. Notably, the (S,N)-rGO/GCE shows the highest current response, which can be attributed to the synergistic effect of (S,N)-co-doping and reduction. The reduction process restores the π-conjugated structure, facilitating faster electron transfer, while heteroatoms create defect sites that enhance electrocatalytic activity. As summarized in Figure 2b, the peak current increases in the order: GCE < GO/GCE < (S,N)-GO/GCE < rGO < (S,N)-rGO/GCE**,** confirming that the (S,N)-rGO-modified electrode exhibits the best electrochemical performance toward SDN detection.

The enhanced electrochemical response of the (S,N)-rGO/GCE can be attributed to the synergistic effects of reduced graphene oxide and nitrogen/sulfur heteroatom doping. The hydrothermal reduction restores the conjugated sp^2^ carbon network, resulting in improved electrical conductivity and accelerated electron-transfer kinetics. Meanwhile, the incorporation of nitrogen and sulfur atoms introduces structural defects and heterogeneous electronic states, which act as additional electroactive sites for the oxidation of SDN. Nitrogen dopants increase the electron density of adjacent carbon atoms, whereas sulfur dopants induce charge polarization and further modulate the local electronic structure. These heteroatom-induced effects facilitate charge transfer across the electrode/electrolyte interface and lower the activation energy for the oxidation reaction. Furthermore, the aromatic structure of SDN can interact with the π-conjugated graphene surface through π–π stacking interactions, while residual oxygen-containing functional groups and heteroatom sites may promote adsorption through hydrogen bonding and electrostatic interactions. Consequently, the combination of improved conductivity, increased density of active sites, and enhanced adsorption affinity results in the significantly higher oxidation current observed at the (S,N)-rGO-modified electrode.

#### Effect of the pH value

**Figure 5:**
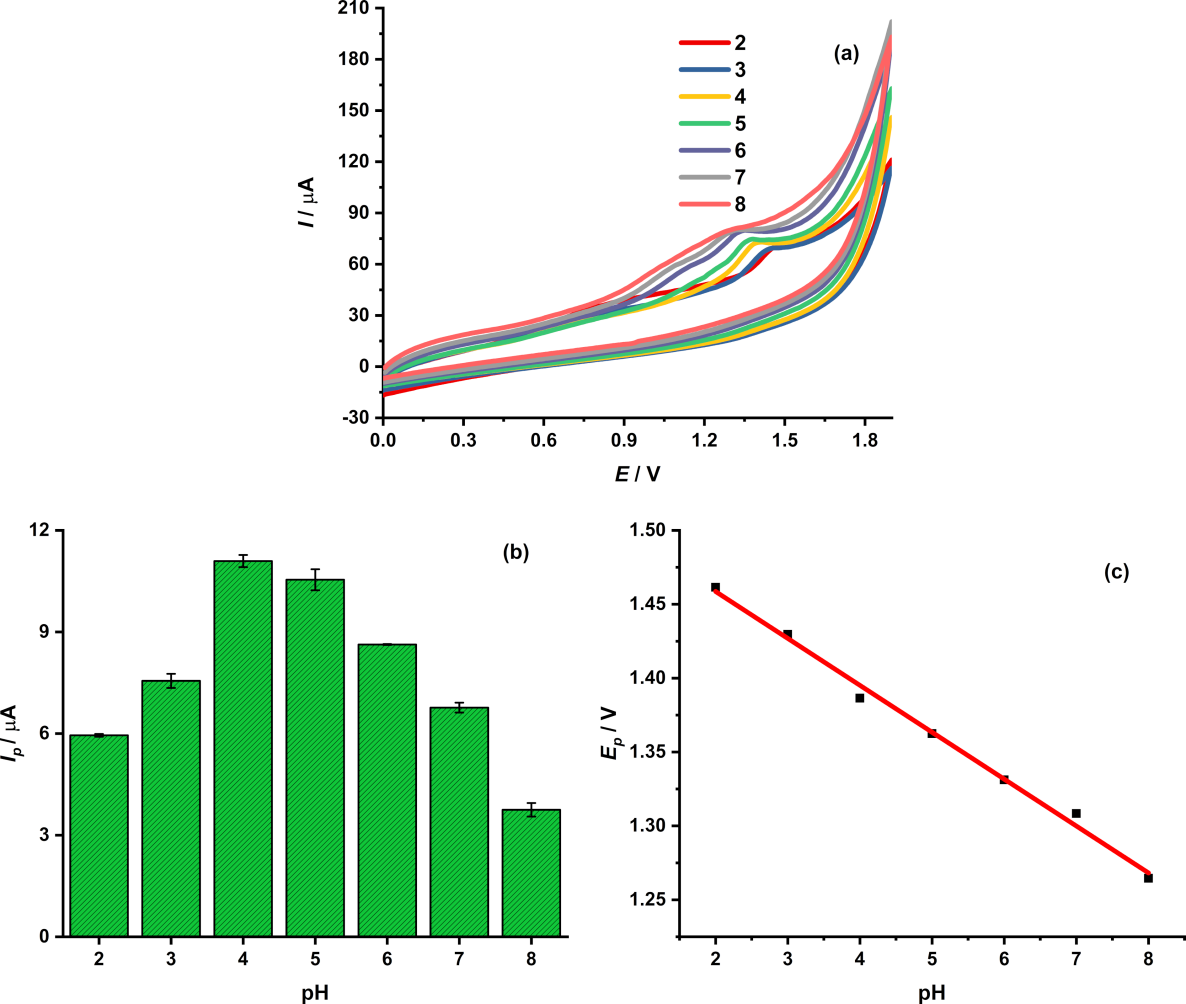
(a) CVs of (S,N)-rGO/GCE in 0.1 M Britton–Robinson buffer at different pH values (2.0–8.0) containing 20 µM SDN at a scan rate of 0.2 V·s^−1^; (b) variation of anodic peak current with pH; (c) dependence of peak potential (*E*_p_) on pH.

The effect of pH on the electrochemical response of SDN at the (S,N)-rGO/GCE was investigated over the pH range of 2.0–8.0 (Figure 5a). The oxidation peak current initially increased with increasing pH, reached a maximum at pH 4.0, and then gradually decreased at higher pH values (Figure 5b). The oxidation peak current initially increased with increasing pH, reached a maximum at pH 4.0, and then gradually decreased at higher pH values (Figure 5b). During the pH study, only a single oxidation peak of SDN was observed in the pH range of 2.0–5.0. However, at pH 6.0–8.0, an additional oxidation peak appeared adjacent to the main SDN oxidation peak, which is likely associated with the formation of oxidation and/or degradation products. Consequently, the CV curves in Figure 5a represent the overall anodic response, whereas the quantitative values shown in Figure 5b were obtained by integrating only the main SDN oxidation peak using Origin software. The contribution of the additional oxidation peak was intentionally excluded from the quantitative analysis. Therefore, although the overall anodic current appears higher at pH 6.0–8.0, the current corresponding solely to the main SDN oxidation peak decreases, resulting in lower peak currents than those observed at pH 4.0 and 5.0. These results indicate that proton participation plays an important role in the electrochemical oxidation of SDN, and therefore pH 4.0 was selected as the optimum supporting electrolyte for subsequent experiments.

As shown in Figure 5c, the oxidation peak potential shifted linearly toward less positive values with increasing pH, following the equation:




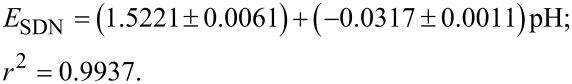




The slope of 0.0317 V·pH^−1^ is close to half of the theoretical Nernstian value of 0.059 V·pH^−1^, suggesting that the number of protons involved in the electrochemical oxidation process is lower than the number of transferred electrons. Based on the Nernst relationship, the proton-to-electron ratio can be estimated to be approximately 0.5, indicating that the number of electrons participating in the oxidation process is likely twice that of protons. However, it should be noted that the reported pH dependence for SDN oxidation varies considerably in the literature. A study [30] has reported slopes close to the theoretical Nernstian value of 0.059 V·pH^−1^, indicating an equal number of protons and electrons participating in the reaction, whereas other [31] has described slopes near half of this theoretical value, corresponding to a proton-to-electron ratio of approximately 1:2. These discrepancies may arise from differences in electrode materials, surface interactions, and oxidation mechanisms. In the present study, the observed deviation may be attributed to the strong electronic interaction between SDN and the conductive (S,N)-rGO surface, together with possible adsorption effects and multistep oxidation pathways occurring at the electrode interface.

#### Scan rate effect

**Figure 6:**
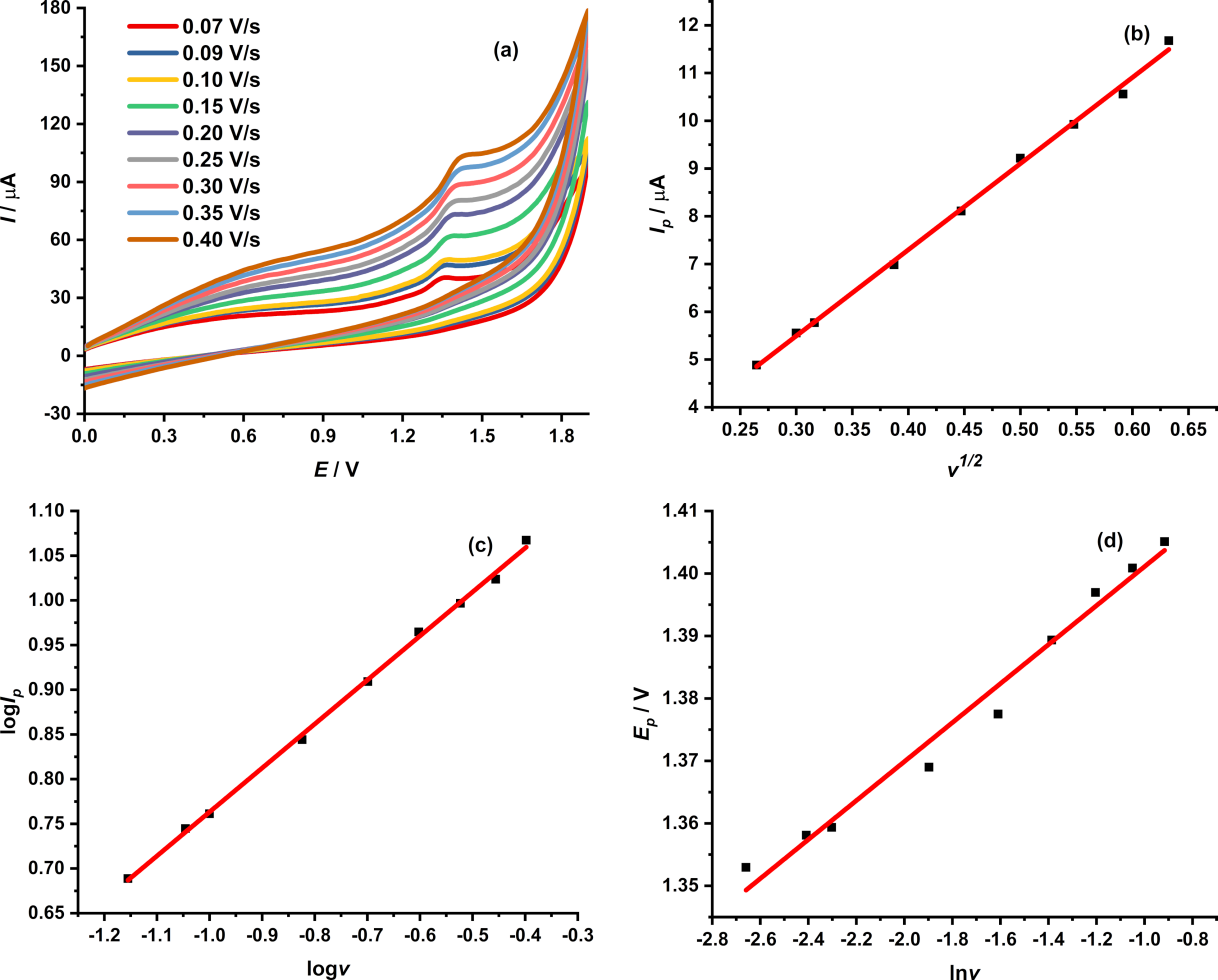
(a) CVs of (S,N)-rGO/GCE in 0.1 M Britton–Robinson buffer (pH 4.0) containing 20 µM SDN at different scan rates (0.07–0.40 V s^−1^); (b) linear relationship between peak current (*I*_p_) and the square root of scan rate (*v*^1/2^); (c) linear plot of log *I*_p_ versus log *v*; (d) dependence of peak potential (*E*_p_) on ln *v*.

The effect of scan rate on the electrochemical behavior of SDN at the (S,N)-rGO/GCE was investigated in the range of 0.07–0.40·V s^−1^ (Figure 6a). The oxidation peak current increased with increasing scan rate, indicating enhanced mass transport toward the electrode surface. As shown in Figure 6b, the peak current exhibited a good linear relationship with the square root of scan rate:




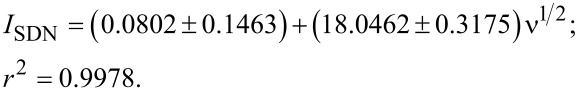




This result indicates that the electrochemical oxidation process is predominantly diffusion-controlled. Further insight was obtained from the log–log relationship (Figure 6c):




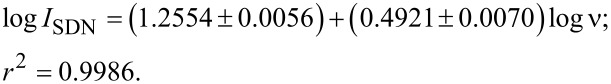




The slope value (≈0.49) is very close to the theoretical value of 0.5 expected for a diffusion-controlled process, further confirming that diffusion is the dominant transport mechanism. Nevertheless, considering the high surface area and π-electron-rich structure of (S,N)-rGO, surface interaction and partial adsorption of SDN on the electrode surface may also contribute to the overall electrochemical response.

For an irreversible electrochemical process, the relationship between peak potential and scan rate is described by the Laviron equation (Figure 6d):




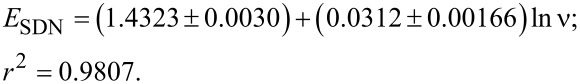




According to Laviron theory [32]:




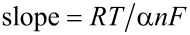




(*R*: molar gas constant (8.314 J·mol^−1^·K^−1^); *T*: the absolute temperature (K); α: the electron-transfer coefficient; *n*: the number of transferred electrons; *F*: the Faraday constant (96485 C·mol^−1^). At 25 °C (*RT*/*F* ≈ 25.7 mV), the slope value of 0.0312 V gives: α*n* = 0.0257/0.0312 ≈ 0.823.

Assuming a typical transfer coefficient of α ≈ 0.5 for an irreversible process [33]:




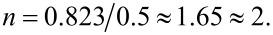




**Figure 7:**
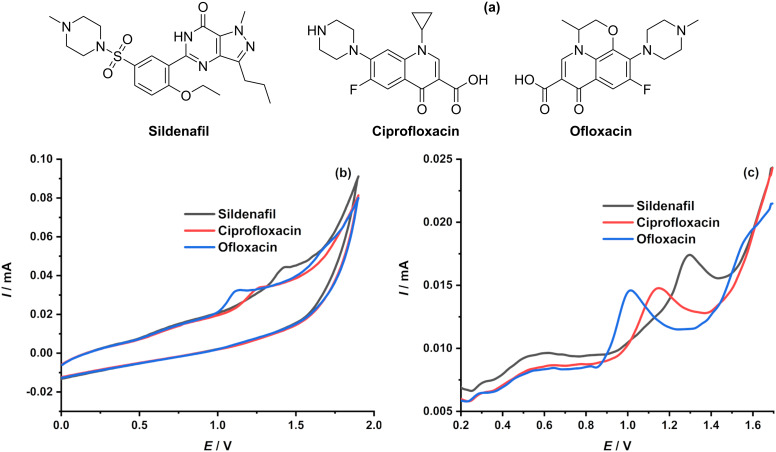
(a) Molecular structures, (b) CVs, and (c) DPVs of SDN, ciprofloxacin, and ofloxacin with concentration of 5.0 µM in 0.1 M BR buffer (pH 4.0).

Thus, the electrooxidation of SDN involves approximately two electrons. Although the exact molecular oxidation mechanism of SDN could not be conclusively established in the present work, the combined pH dependence, scan-rate investigation, and Laviron analysis consistently indicate the participation of approximately two electrons and one proton in the overall irreversible oxidation process. In addition, the cyclic voltammetric responses of SDN were compared with those of ciprofloxacin and ofloxacin (Figure 7), which are also nitrogen-containing heterocyclic pharmaceuticals. Ciprofloxacin and ofloxacin exhibited relatively sharp and well-defined oxidation peaks, whereas SDN showed a broader anodic response with less-defined peak characteristics. These observations suggest that the electrooxidation of SDN at the (S,N)-rGO/GCE may involve multistep electron-transfer reactions and/or adsorbed intermediates rather than a simple proton-coupled electron-transfer process. Furthermore, the presence of tertiary nitrogen centers and a flexible heterocyclic framework in SDN may promote strong interaction with the π-conjugated conductive surface, thereby altering the apparent proton participation during oxidation and contributing to the lower *E*_p_–pH slope observed experimentally. Based on the *E*_p_–pH relationship and Laviron analysis, the electrooxidation of SDN is hypothesized to involve an apparent two-electron/one-proton (2e^−^/1H^+^) transfer process. However, this interpretation should be considered a plausible hypothesis rather than a definitive reaction mechanism, as the oxidation of SDN is known to involve complex multistep electron-transfer reactions. Moreover, adsorption of SDN and/or its oxidation intermediates on the (S,N)-rGO surface may influence the electrochemical response and the apparent kinetic parameters. Therefore, further mechanistic investigations, including in situ spectroelectrochemical measurements or identification of oxidation products, would be valuable for elucidating the detailed oxidation mechanism.

#### Optimizing operational parameters

The experimental parameters were optimized to achieve the best analytical performance (Supporting Information File 1, Figures S1–S4). The optimal conditions were determined as follows: accumulation potential of 0.4 V, accumulation time of 40 s, pulse amplitude of 0.10 V, and potential step of 0.007 V.

#### Linear range and limit of detection

**Figure 8:**
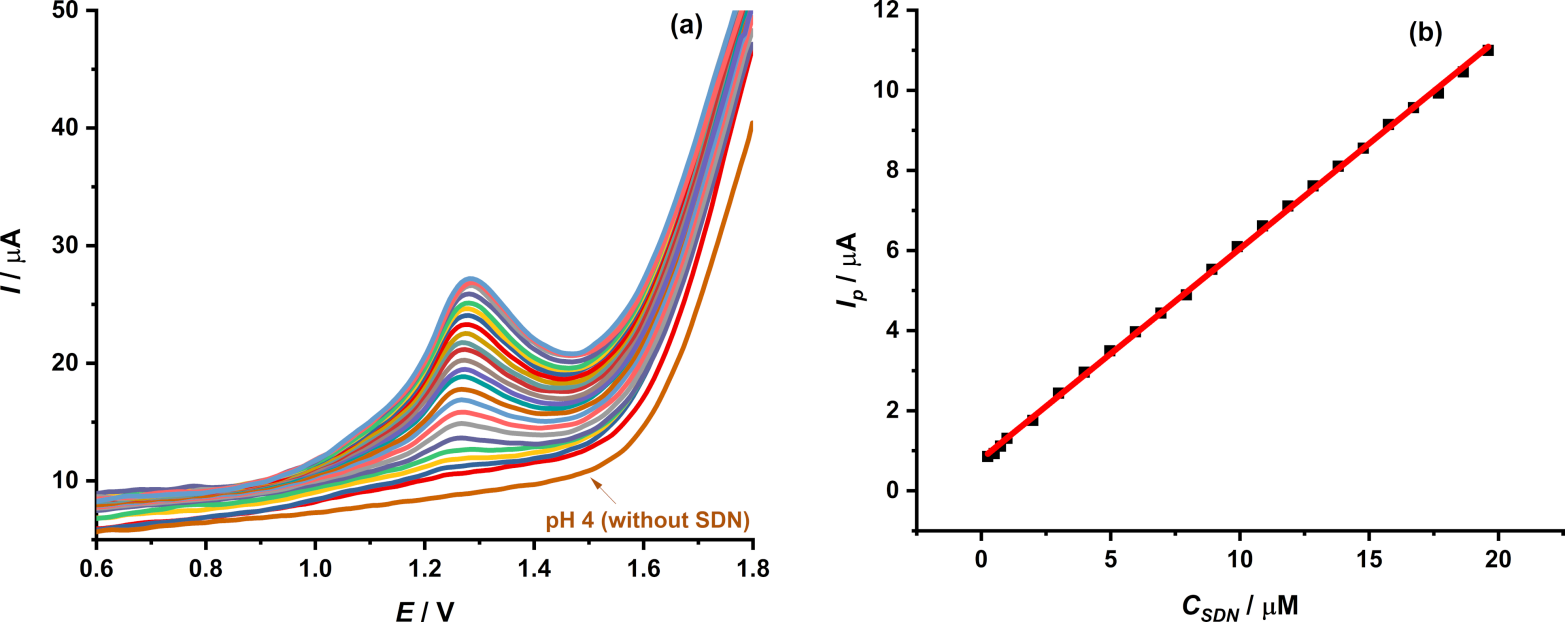
(a) DPV curves of (S,N)-rGO/GCE in 0.1 M Britton–Robinson buffer (pH 4.0) for different concentrations of *C*_SDN_ (0.25–19.61 µM); (b) corresponding calibration plot of peak current versus SDN concentration.

The analytical performance of the proposed sensor was evaluated using DPV under the optimized conditions. As shown in Figure 8a, the oxidation peak current increases progressively with increasing SDN concentration from 0.25 to 19.61 µM. A good linear relationship between peak current and SDN concentration was obtained over this range (Figure 8b), described by the following equation:




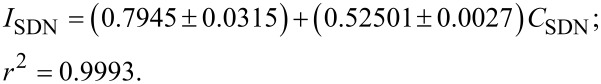




The LOD and the LOQ, calculated according to the criteria of 3S/b and 10S/b, respectively [34–35] were determined to be 0.49 and 1.61 µM. To further improve sensitivity at low concentrations, a narrower linear range (0.25–4.98 µM) was considered using eight concentration points near the origin. In this range, LOD and LOQ were significantly improved to 0.24 and 0.78 µM, respectively, demonstrating the enhanced detection capability of the sensor at trace levels.

As summarized in Table 2, the proposed (S,N)-rGO/GCE sensor exhibits a lower detection limit than most previously reported electrochemical sensors for SDN determination, while providing a wide linear range and satisfactory applicability for the analysis of pharmaceutical formulations.

**Table 2:** Comparison of the analytical performance of electrochemical sensors for the determination of SDN.

Electrode	Method	Linear range (µM)	LOD (µM)	Application	Ref.

SPGCE	SWV	1.0–14.0	0.055	pharmaceutical and urine samples	[36]
SPCE	SWV	1.0–20.0	0.20	commercial and adulterated tablets	[37]
BDDE	DPV	0.730–7.3	0.64	pharmaceutical formulations	[38]
ABPUE-AgNP	DPV	1.0–10.0	0.89	pharmaceutical formulations and synthetic urine	[31]
Paraff-GE	SWV	25–250	9.0	pharmaceutical formulations	[39]
RuQT	DPV	12.5–500.0	10.7	pharmaceutical formulations	[40]
(S,N)-rGO/GCE	DPV	0.25–19.61	0.24	pharmaceutical formulations	this work

#### Repeatability, reproducibility, and long-term stability

**Figure 9:**
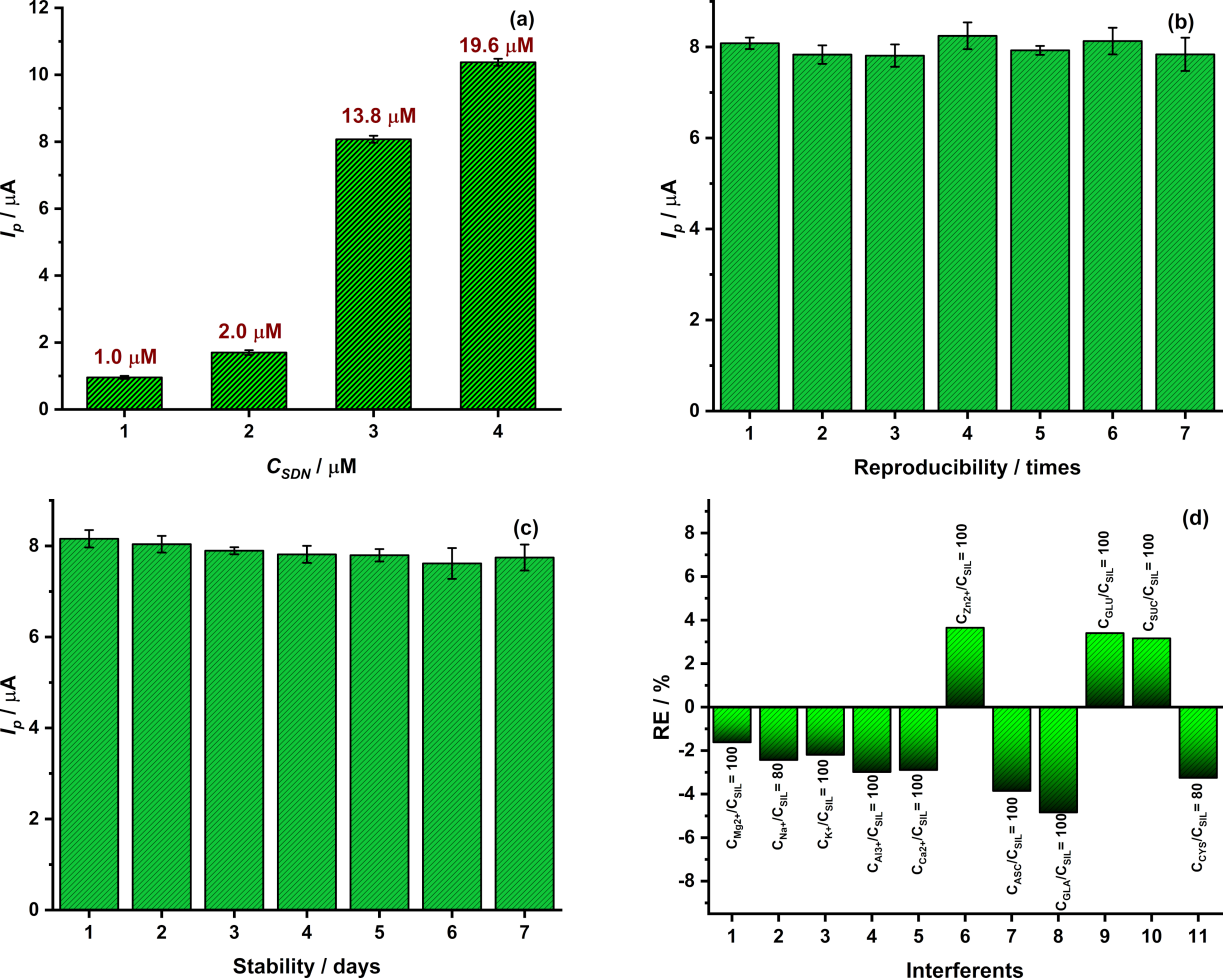
(a) Variation of peak current (*I*_p_) for SDN at four concentrations (1.0, 2.0, 13.8, and 19.6 µM) recorded at the (S,N)-rGO/GCE in 0.1 M Britton–Robinson buffer (pH 4.0); (b) repeatability of the peak current at a fixed concentration (*C*_SDN_ = 13.8 µM) measured over seven successive determinations using the same electrode preparation procedure; (c) long-term stability of the sensor evaluated over seven consecutive days; (d) relative error (RE%) caused by interfering substances at different concentration ratios.

Intra-day repeatability (within-electrode precision) of the proposed electrochemical method was evaluated at four representative concentration levels (1.0, 2.0, 13.8, and 19.6 µM) using replicate measurements (*n* = 3) (Figure 9a and Supporting Information File 1, Figure S5). At 1.0 µM, the peak current was 0.9585 ± 0.0469 µA, corresponding to an RSD of 4.90%, which is lower than the acceptable criterion of 0.5RSD_Horwitz_ = 8.50%. Similarly, at 2.0 µM, the peak current was 1.6990 ± 0.0732 µA with an RSD of 4.31%, also below the threshold (0.5RSD_Horwitz_ = 7.66%). At higher concentrations, improved precision was observed, with RSD values of 1.30% and 1.02% for 13.8 and 19.6 µM, respectively, both significantly lower than the corresponding 0.5RSD_Horwitz_ values (5.73% and 5.43%) [41]. The electrode-to-electrode reproducibility of the sensor was further assessed using seven independently prepared electrodes at a fixed concentration of 13.8 µM (Figure 9b). The peak current was 7.9800 ± 0.1716 µA with an RSD of 2.15%, indicating good fabrication reproducibility. In addition, the long-term stability of the modified electrode was evaluated over seven consecutive days (Figure 9c). The average peak current was 7.8657 ± 0.1828 µA with an RSD of 2.32%, and the signal decreased by only 5.06% after seven days, demonstrating good storage stability. Overall, these results confirm that the proposed (S,N)-rGO/GCE sensor provides good precision, reproducibility, and stability, making it suitable for reliable electrochemical analysis.

#### Selectivity

The selectivity of the proposed electrochemical sensor was evaluated by investigating the effect of common inorganic ions and organic substances that may coexist with SDN (Supporting Information File 1, Table S1). Various potential interferents, including Mg^2+^, Na^+^, K^+^, Al^3+^, Zn^2+^, and Ca^2+^, as well as organic compounds such as ascorbic acid (ASC), glutamic acid (GLA), glucose (GLU), sucrose (SUC), and ʟ-cysteine (CYS), were evaluated at concentration ratios ranging from 0 to 100-fold relative to SDN, except for Na^+^ and sucrose (SUC), which were investigated up to 80-fold (Figure 9d). As shown in Figure 9d, the presence of these interfering species caused only minor variations in the peak current of SDN. The relative error (RE) values were generally within ±5%, even at high interferent-to-analyte ratios (up to 100). In particular, most inorganic ions exhibited negligible interference, with RE values typically below ±3%. Organic compounds such as glucose and sucrose showed slight positive deviations, while ascorbic acid and ʟ-cysteine resulted in small negative deviations, which can be attributed to their electroactive nature. However, these effects remained within acceptable analytical limits. According to commonly accepted criteria (|RE| ≤ 5%), these results demonstrate that the proposed (S,N)-rGO/GCE sensor possesses good selectivity for the determination of *C*_SDN_ in the presence of potential interfering substances. The high selectivity of the sensor suggests its strong potential for application in complex real samples.

#### Analysis of real sample

The applicability of the proposed (S,N)-rGO/GCE sensor was evaluated by determining SDN in commercial pharmaceutical samples using the DPV method. Three different products were analyzed, and the results are summarized in Table 3. The measured concentrations of *C*_SDN_ in the electrochemical cell were found to be 4.07 ± 0.07, 4.22 ± 0.08, and 4.05 ± 0.08 µM for samples 1–3, respectively. These values were converted to the corresponding SDN content per dosage unit, yielding 48.4, 102.5, and 47.6 mg, respectively. The analytical accuracy of the proposed sensor was evaluated using two complementary approaches. First, recovery experiments were carried out by the standard addition method, yielding satisfactory recoveries ranging from 97.6% to 103.5%. Second, the results obtained by the proposed DPV method were compared with those obtained using the reference HPLC method. No statistically significant difference was observed between the two methods, confirming the accuracy, reliability, and practical applicability of the developed electrochemical sensor for the determination of SDN in pharmaceutical formulations.

**Table 3:** Determination of SDN in commercial pharmaceutical samples using the proposed DPV method and comparison with the HPLC method.

Sample name	Found (µM)^a^ ± SD (µM)	SDN (mg per unit, DPV)	SDN (mg per unit, HPLC)	Added (µM)	Found after spiking (µM) ± SD^a^ (µM)	Recovery (%)

1	4.07 ± 0.07	48.4	51.5	1.00	5.06 ± 0.06	98.1
2	4.22 ± 0.08	102.5	102.0		5.36 ± 0.14	103.5
3	4.05 ± 0.08	47.6	48.7		5.06 ± 0.06	97.6

^a^Concentration calculated in the electrochemical cell.

Although the developed (S,N)-rGO/GCE sensor demonstrated excellent analytical performance for the determination of SDN in pharmaceutical formulations, several limitations should be acknowledged. The present study focused exclusively on pharmaceutical samples, and therefore additional validation in more complex biological and environmental matrices is still required. Furthermore, while satisfactory stability was observed over a seven-day period, longer-term operational stability under routine analytical conditions remains to be investigated. The simple drop-casting fabrication procedure may also introduce slight variations in the modified film between independently prepared electrodes. In addition, although the sensor exhibited good selectivity toward the investigated interferents, structurally related electroactive compounds at relatively high concentrations could potentially influence the analytical response. Future work will focus on improving electrode fabrication reproducibility, extending the sensor lifetime, and evaluating its applicability in complex real-world samples.

## Conclusion

In this study, an efficient electrochemical sensor based on a glassy carbon electrode modified with (S,N)-doped reduced graphene oxide ((S,N)-rGO) was successfully developed for the determination of SDN. The successful incorporation of nitrogen and sulfur into the graphene structure was confirmed by multiple characterization techniques, resulting in enhanced structural defects and improved electrochemical properties. The (S,N)-rGO/GCE exhibited excellent electrocatalytic activity toward SDN oxidation, enabling sensitive detection with a wide linear range (0.25–4.98 µM), a low limit of detection (0.24 µM), and a limit of quantification of 0.78 µM. The sensor also demonstrated good repeatability, reproducibility, and long-term stability, together with high selectivity in the presence of potential interfering species. Importantly, the proposed method was successfully applied to the analysis of pharmaceutical samples, and the results were in good agreement with those obtained by HPLC, confirming its accuracy and reliability. These findings indicate that the (S,N)-rGO-based electrochemical sensor provides a simple, cost-effective, and reliable alternative for the routine determination of SDN in real samples.

The proposed (S,N)-rGO-functionalized sensing platform offers several advantages, including a simple and low-cost fabrication process, enhanced electron-transfer capability, high electrocatalytic activity, satisfactory selectivity, and reliable determination of SDN in pharmaceutical formulations. Compared with conventional chromatographic techniques, the proposed electrochemical sensor provides rapid analysis with minimal sample preparation and lower instrumentation cost. Nevertheless, the current study is limited to pharmaceutical samples, and further investigations are required to evaluate long-term operational stability and its applicability to more complex biological and environmental matrices.

## Supporting Information

File 1 Additional figures.

## Data Availability

All data that supports the findings of this study is available in the published article and/or the supporting information of this article.
