# Supplementary material for: Electrochemical sensing of sildenafil in pharmaceutical formulations using an (S,N)-doped reduced graphene oxide-modified electrode
Source: Beilstein J Nanotechnol. 2026 Aug 11;17:1087–102. doi: 10.3762/bjnano.17.74 (PMC13478895; doi:10.3762/bjnano.17.74)
Supplement: Supplementary file 1 [file Beilstein_J_Nanotechnol-17-1087-s001.pdf]

BEILSTEIN JOURNAL OF NANOTECHNOLOGY

Supporting Information

for

Electrochemical sensing of sildenafil in pharmaceutical formulations using an (S,N)-doped reduced graphene oxide-modified electrode

Nguyen Quang Man, Ngo Thi Thanh Xuan, Vo Thi Khanh Ly, Le Van Thanh Son, Le Duc Anh, Le Thi Hong Phong, Nguyen Hai Phong, Pham Khac Lieu, Le Lam Son and Dinh Quang Khieu

Beilstein J. Nanotechnol. **2026,** *17,* 1087–1102. doi:10.3762/bjnano.17.74

Additional figures

License and Terms: This is a supporting information file under the terms of the Creative Commons Attribution License (https://creativecommons.org/licenses/by/4.0). Please note that the reuse, redistribution and reproduction in particular requires that the author(s) and source are credited and that individual graphics may be subject to special legal provisions.
The license is subject to the *Beilstein Journal of Nanotechnology* terms and conditions: (https://www.beilstein-journals.org/bjnano/terms)

The optimal experimental conditions were selected by considering not only the peak current but also the peak shape, baseline stability, repeatability, and overall analytical reliability. When several conditions produced comparable peak-current responses, the condition yielding the most stable and well-defined oxidation peak was selected.

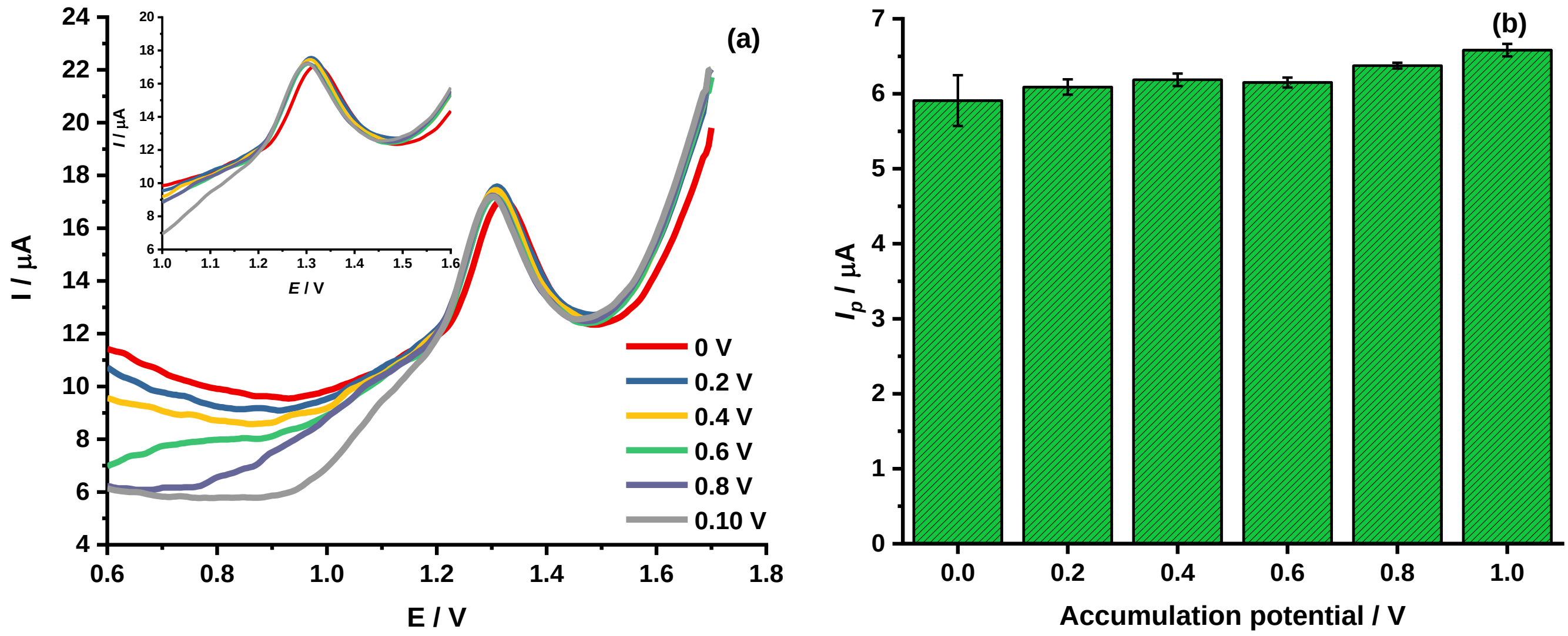

Figure S1: (a) Differential pulse voltammetry (DPV) curves of (S,N)-rGO/GCE recorded in 0.1 M Britton–Robinson buffer (pH 4.0) at a fixed SIL concentration of 20 µM over the potential range from 0.0 to +1.8 V (Experimental conditions: accumulation time, 10 s; pulse amplitude, 0.05 V; step potential, 0.005 V); (b) Variation of peak current with accumulation potential.

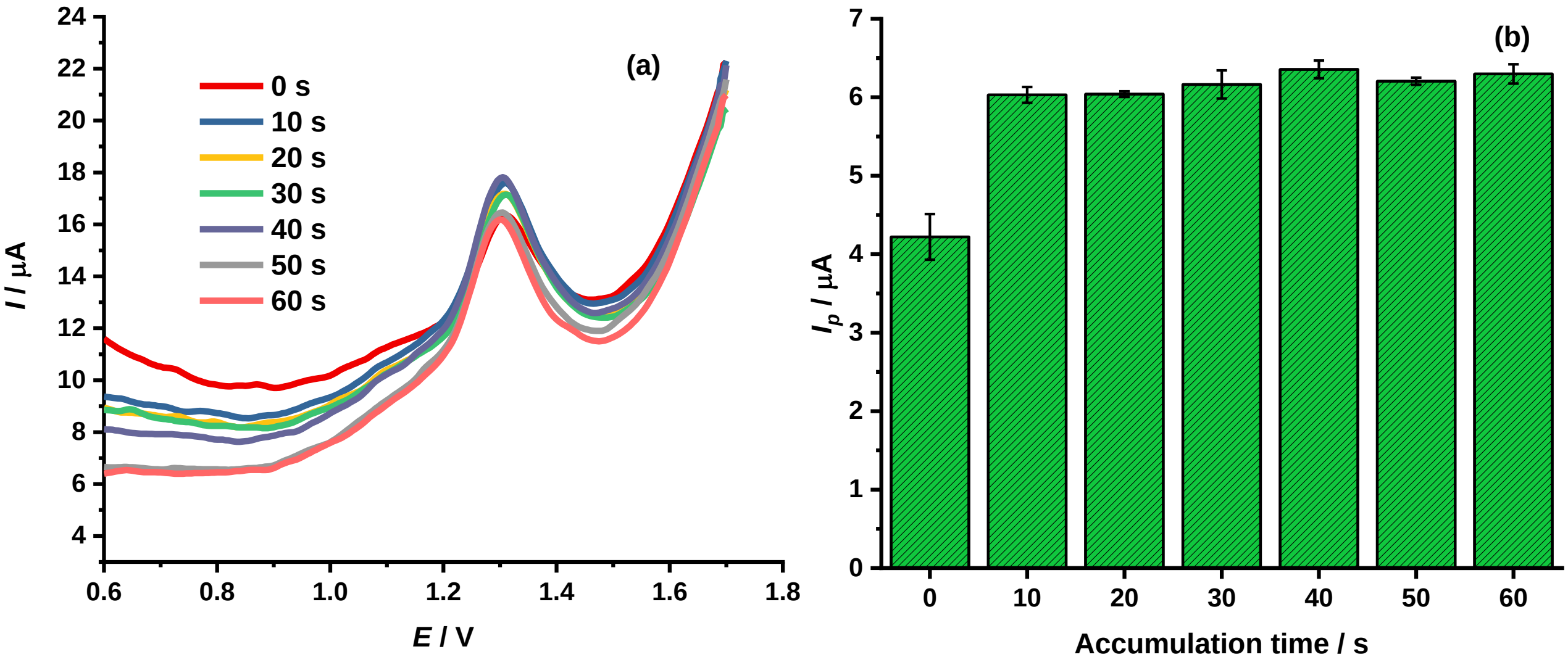

Figure S2: (a) DPVcurves of (S,N)-rGO/GCE recorded in 0.1 M Britton–Robinson buffer (pH 4.0) at a fixed SIL concentration of 20 µM, with accumulation time varying from 0 to 60 s (Experimental conditions: accumulation potential, 0.4 V; pulse amplitude, 0.05 V; step potential, 0.005 V); (b) Variation of peak current with accumulation time.

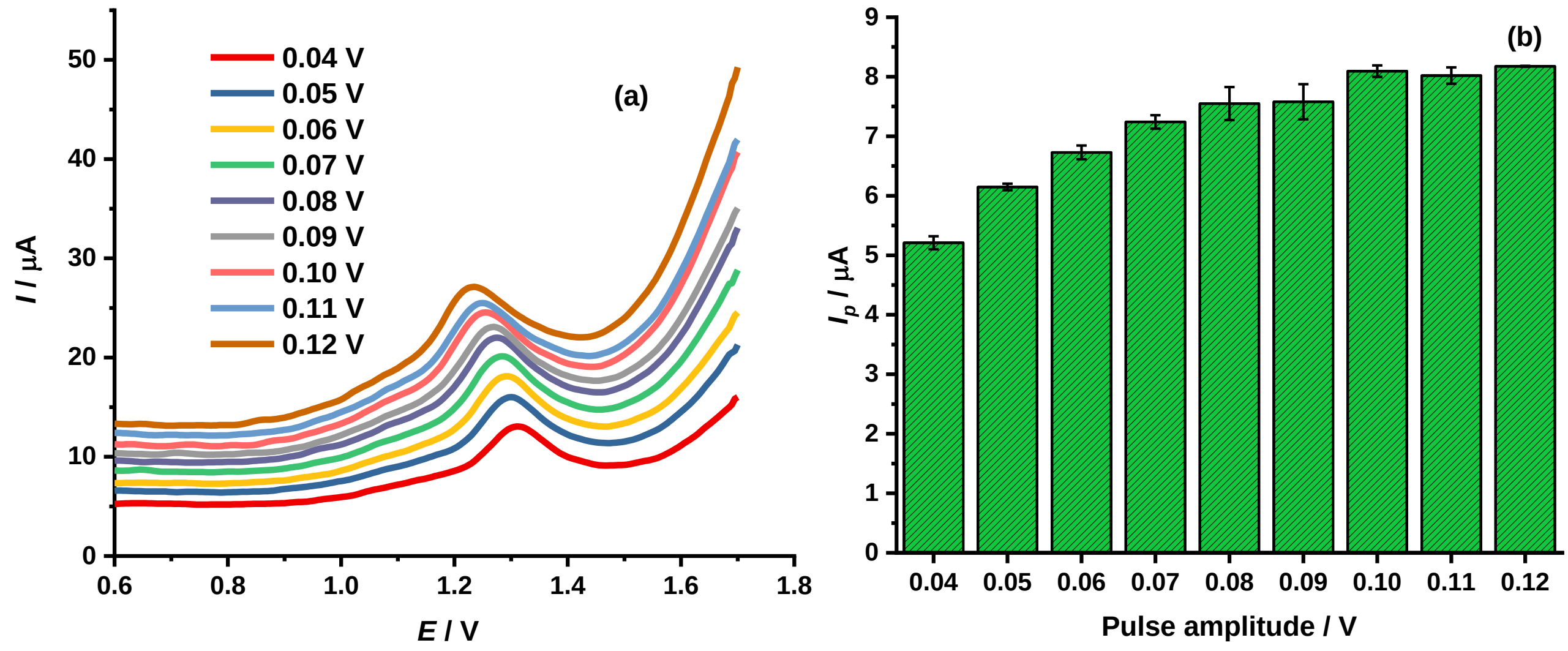

Figure S3: (a) DPV curves of (S,N)-rGO/GCE recorded in 0.1 M Britton–Robinson buffer (pH 4.0) at a fixed SIL concentration of 20 μM, with pulse amplitude varying from 0.04 to 0.12 V (Experimental conditions: accumulation potential, 0.4 V; accumulation time, 40 s; step potential, 0.005 V); (b) Variation of peak current with pulse amplitude.

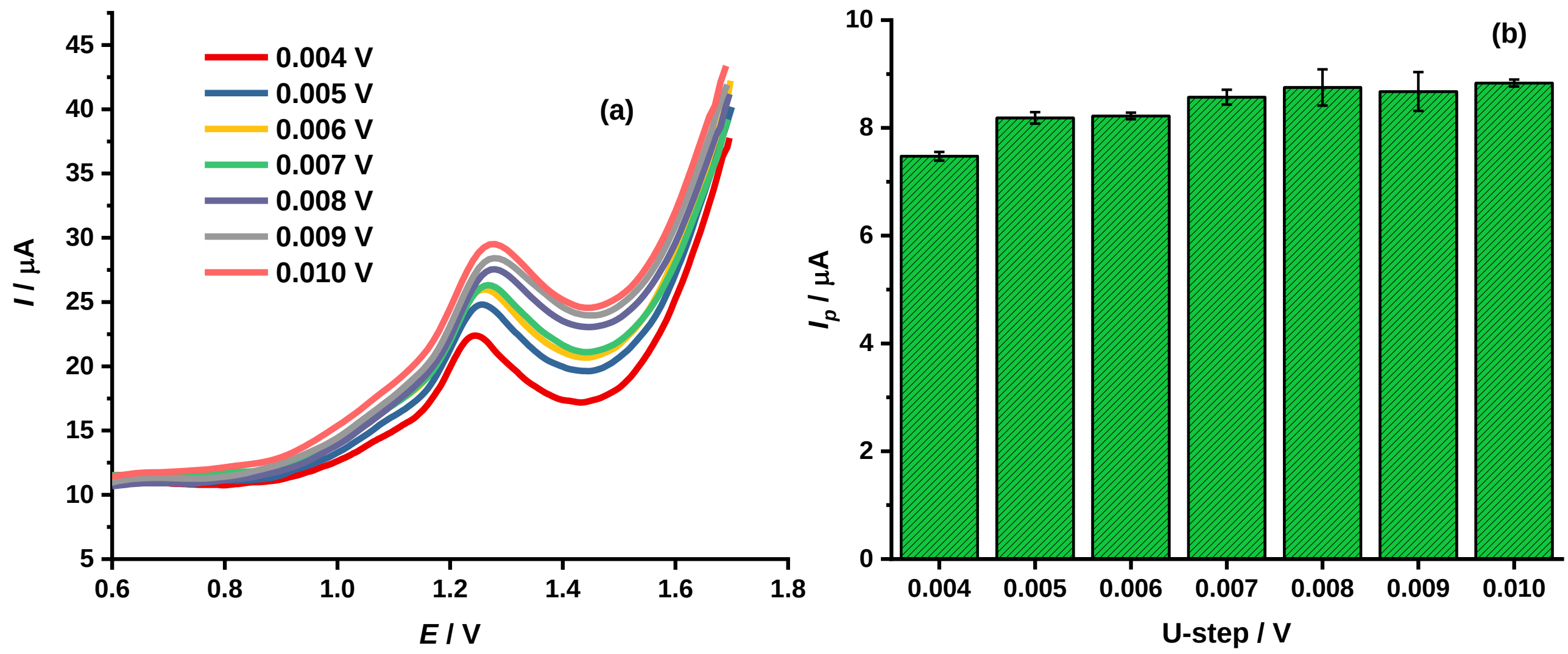

Figure S4: (a) DPV curves of (S,N)-rGO/GCE recorded in 0.1 M Britton–Robinson buffer (pH 4.0) at a fixed SIL concentration of 20 μM, with step potential varying from 0.004 to 0.010 V (Experimental conditions: accumulation potential, +0.4 V; accumulation time, 40 s; pulse amplitude, 0.10 V); (b) Variation of peak current with step potential.

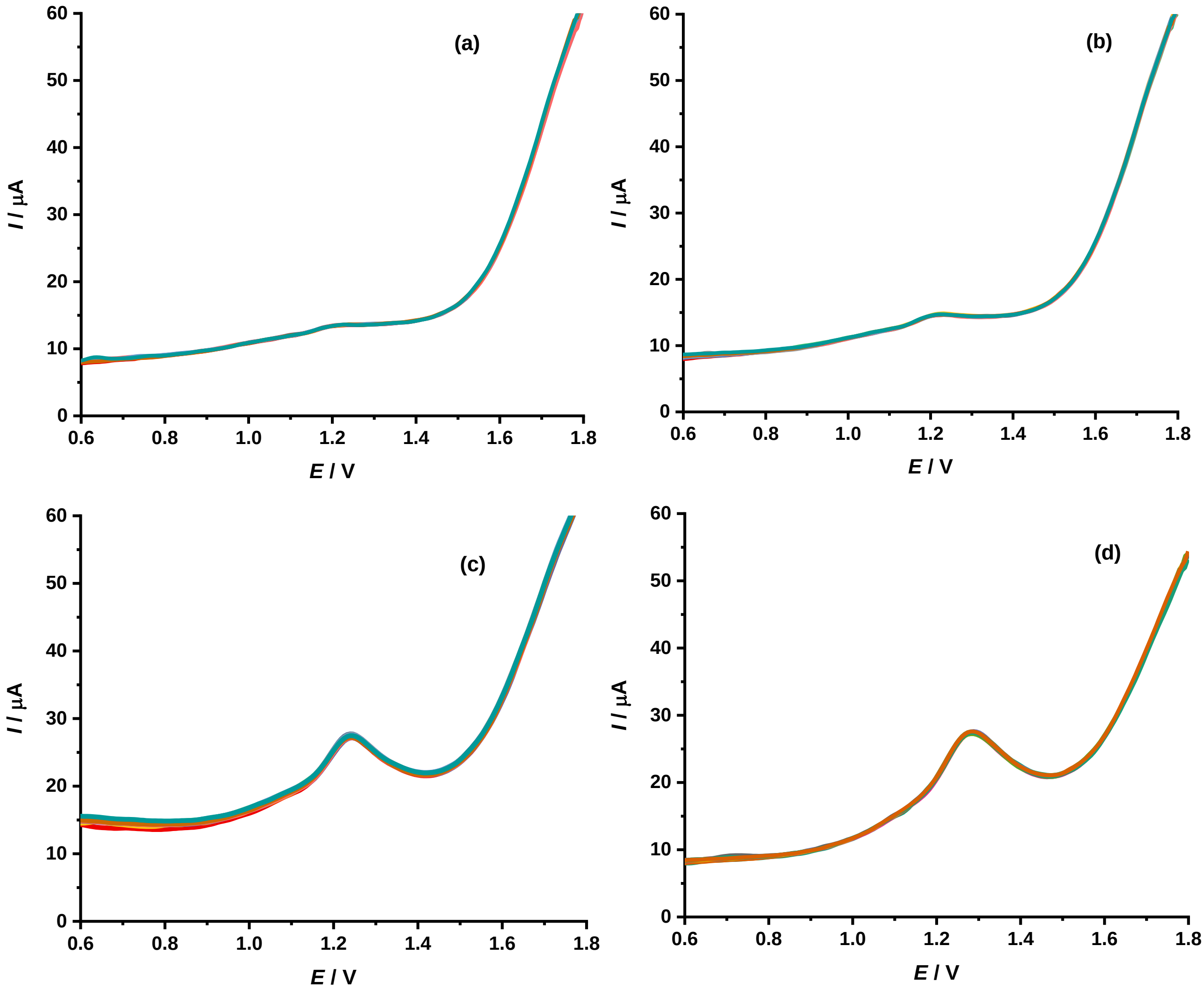

Figure S5: DPV curves of (S,N)-rGO/GCE recorded in 0.1 M Britton–Robinson buffer (pH 4.0) at different SIL concentrations: (a) 1.0 µM, (b) 2.0 µM, (c) 13.8 µM, and (d) 19.6 µM.

Table S1: Effect of potential interfering organic and inorganic substances on the peak current response of SIL at the (S,N)-rGO/GCE.

**1. $MgCl_2$**			**2. Na_2SO_4**		
**C_{NaCl}/C_{SIL}**	**Ip (µA)**	**Re (%)**	**C_{K2SO4}/C_{SIL}**	**Ip (µA)**	**Re (%)**
0	8.01	**0.00**	0	8.05	**0.00**
20	7.99	**-0.25**	20	8.04	**-0.17**
40	7.97	**-0.53**	40	7.92	**-1.66**
60	7.95	**-0.72**	60	7.88	**-2.11**
80	7.92	**-1.09**	80	7.86	**-2.43**
100	7.88	**-1.62**	100	7.61	**-5.50**
**3. KNO_3**			**4. $AlCl_3$**		
**C_{MgSO4}/C_{SIL}**	**Ip (µA)**	**Re (%)**	**$C_{Ca(HPO4)2}/C_{SIL}$**	**Ip (µA)**	**Re (%)**
0	7.87	**0.00**	0	7.74	**0.00**
20	7.87	**-0.04**	20	7.74	**-0.04**
40	7.83	**-0.51**	40	7.70	**-0.56**

60	7.80	**-0.95**	60	7.53	**-2.76**
80	7.76	**-1.44**	80	7.54	**-2.69**
100	7.70	**-2.19**	100	7.51	**-2.98**
**5- $Ca(HPO_4)_2$**			**6- $Zn(NO_3)_2$**		
**C_{ZnCl2}/C_{SIL}**	**Ip (μA)**	**Re (%)**	**C_{NH4NO3}/C_{SIL}**	**Ip (μA)**	**Re (%)**
0	7.51	**0.00**	0	7.46	**0.00**
20	7.47	**-0.53**	20	7.44	**-0.28**
40	7.45	**-0.75**	40	7.52	**0.77**
60	7.41	**-1.33**	60	7.57	**1.51**
80	7.39	**-1.60**	80	7.65	**2.58**
100	7.29	**-2.89**	100	7.73	**3.65**
**7- Ascorbic acid**			**8-Glutamic acid**		
**C_{ASC}/C_{SIL}**	**Ip (μA)**	**Re (%)**	**C_{GLA}/C_{SIL}**	**Ip (μA)**	**Re (%)**
0	7.68	**0.00**	0	7.55	**0.00**
20	7.54	**-1.85**	20	7.49	**-0.79**
40	7.49	**-2.51**	40	7.49	**-0.78**
60	7.44	**-3.11**	60	7.46	**-1.22**
80	7.41	**-3.50**	80	7.31	**-3.21**
100	7.39	**-3.85**	100	7.19	**-4.84**
**9-D-Glucose**			**10-D-Sucrose**		
**C_{GLU}/C_{SIL}**	**Ip (μA)**	**Re (%)**	**C_{SUC}/C_{SIL}**	**Ip (μA)**	**Re (%)**
0	7.33	**0.00**	0	7.44	**0.00**
20	7.43	**1.37**	20	7.45	**0.16**
40	7.43	**1.34**	40	7.55	**1.50**
60	7.45	**1.57**	60	7.53	**1.31**
80	7.54	**2.87**	80	7.63	**2.66**
100	7.58	**3.40**	100	7.67	**3.16**
**11- L-cysteine**					
**C_{CYS}/C_{SIL}**	**Ip (μA)**	**Re (%)**			
0	7.70	**0.00**			
20	7.62	**-1.04**			
40	7.57	**-1.73**			
60	7.51	**-2.55**			
80	7.45	**-3.25**			
100	7.28	**-5.50**			
